# What's new is older

**DOI:** 10.7554/eLife.00605

**Published:** 2013-03-20

**Authors:** Lara M Rangel, Howard Eichenbaum

**Affiliations:** 1 **Lara M Rangel** is at the Center for Memory and Brain, Boston University, Boston, United Statesrangel@bu.edu; 2 **Howard Eichenbaum** is at the Center for Memory and Brain, Boston University, Boston, United Stateshbe@bu.edu

**Keywords:** learning and memory, dentate gyrus, pattern separation, CA1, hippocampus, neuroscience, Mouse

## Abstract

Distinct populations of active cells in the dentate gyrus of the hippocampus may facilitate the unique encoding of changes in the environment.

**Related research article** Deng W, Mayford M, Gage FH. 2013. Selection of distinct populations of dentate granule cells in response to inputs as a mechanism for pattern separation in mice. *eLife* **2**:e00312. doi: 10.7554/eLife.00312

**Image** Coloured reporter constructs reveal activated cells in the dentate gyrus

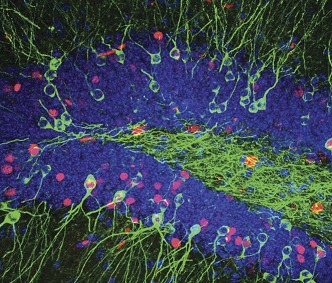

Distinguishing between memories of very similar events can be difficult even if the events occurred only recently. Memory for personal experiences is mediated by a region of the brain called the hippocampus, and one subregion in particular, known as the dentate gyrus, is thought to contribute to the encoding of similar events as distinct memories. This process is called pattern separation, but how it is accomplished at the cellular level, and whether the dentate gyrus also participates in the retrieval of such memories, is uncertain. Moreover, the dentate gyrus is notable for being one of only two regions in the adult brain in which new neurons are produced through a process called neurogenesis. But whether and how neurogenesis is involved in keeping similar memories distinct is again unclear.

The dentate gyrus is the first component of the trisynaptic circuit, which is one route along which information is relayed through the hippocampus. Excitatory input from the cerebral cortex arrives at the dentate gyrus via the perforant pathway. The axons of granule cells within the dentate gyrus then project to another subregion called CA3, where they form synapses with pyramidal neurons. The axons of these pyramidal cells in turn project to the CA1 subregion, which provides the final output of the hippocampus back to cortical areas. By detecting the expression of immediate early genes (IEGs), which are rapidly and transiently transcribed whenever a cell is activated, it is possible to map the population of neurons within each subregion that are active at any given time. Some IEG experiments, and also computational models, have suggested that the dentate gyrus encodes similar events as distinct by recruiting different populations of granule cells for each memory (Treves and Rolls, 1992; O'Reilly and McClelland, 1994; Chawla et al., 2005; Rolls and Kesner, 2006; Aimone et al., 2009; Liu et al., 2012). However, other IEG and *in vivo* electrophysiological experiments have instead indicated that the dentate gyrus recruits overlapping populations of granule cells to encode different events, and distinguishes between the events using methods such as changes in firing rate (Leutgeb et al., 2007; Alme et al., 2010; Rennó-Costa et al., 2010). Now writing in *eLife*, Wei Deng and Fred Gage at The Salk Institute for Biological Studies, plus Mark Mayford at the Scripps Research Institute, combine cutting edge genetic and IEG techniques to clarify the specific circumstances under which different populations of granule cells are activated (Deng et al., 2013).

It has long been hypothesized that one of the functions of the dentate gyrus is to decorrelate activity patterns in the CA3 subregion; that is, to ensure that output patterns of neuronal activity in CA3 are less similar to one another than the input patterns of neuronal activity that entered the dentate gyrus (Treves and Rolls, 1992; O'Reilly and McClelland, 1994). This role is supported by the presence of sparse but powerful inputs from the dentate gyrus to CA3 and low observed numbers of active granule cells (Jung and McNaughton, 1993; Chawla et al., 2005). Since the dentate gyrus is a site of adult neurogenesis, it is important to take into account the unique physiology of immature adult-born cells in theories of dentate function. One hypothesis is that these cells show a transient developmental window during which they respond similarly to any event (Aimone et al., 2006), but, upon maturation, they become a population of granule cells that are activated only upon re-exposure to experiences similar to those that occurred during their development (Tashiro et al., 2007; Aimone et al., 2009). Thus, it was long anticipated that experiments examining the effects on dentate gyrus activity of changing spatial, temporal or other learning conditions would demonstrate the recruitment of distinct populations of cells.

To examine this hypothesis, Deng et al. placed mice in a highly familiar environment and compared the neuronal populations that were active over successive time windows. They then compared the initial activity to that observed during a subsequent exposure to the same environment (with or without the addition of a fearful event), to a new environment, or to a modified version of the familiar environment (Figure 1A,B,C respectively). To identify the neuronal populations that encoded each experience, they used a transgenic mouse in which doxycycline (dox) regulated the expression of a reporter construct. Exposing the mice to the familiar environment in the absence of dox led to any cells active during the initial encoding being labeled with the construct (Figure; top row, green labeled cells). After re-administering dox to suppress subsequent expression of the reporter construct, Deng et al. gave the mice a second experience and detected the cells that were active during this experience by quantifying the expression of the IEGs cfos or EGR1 (middle row, red labeled cells). Cells that were active during both experiences expressed the reporter construct as well as the IEGs (bottom row, yellow labeled cells).

**Figure 1.**
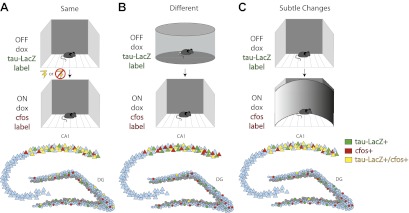
Deng et al. investigate differential population coding in the dentate gyrus by comparing cells activated during two successive experiences. **Top row:** Transgenic mice in which the expression of a green reporter construct (tau-LacZ) was under the control of doxycycline (dox) were exposed to a highly familiar environment. Dox was withdrawn during exposure, such that any cells that were active during the experience were labeled green. **Middle row:** Dox was then re-administered prior to exposure to the same environment again with or without the addition of an electric shock between exposures (**A**); to a very different environment (**B**); or to a similar environment (**C**). Any cells that were activated during the second experience were labeled with the immediate early gene cfos (red). **Bottom row:** Summary figure characterizing the numbers of cells activated in the first (*green*) or second (*red*) experience, or in both experiences (*yellow*). Repeated exposures to the same environment (with or without an electric shock) activated largely overlapping cell populations in the CA1 subregion, but had no effect on which cells were activated in the dentate gyrus (**A**). Exposure to two very different environments activated largely distinct cell populations in the dentate gyrus, but did not affect which cells were activated in CA1 (**B**). Exposure to two similar environments activated largely overlapping populations of CA1 neurons, but largely distinct populations of dentate gyrus granule cells (**C**). In all three conditions, most of the cells that were activated in the dentate gyrus were mature granule cells (locations of inactive mature cells indicated in blue) rather than immature adult-born neurons (locations of inactive adult-born cells indicated in gray).

Deng et al. observed less overlap between the populations of granule cells that were active during exposure to two different environments, or to an initial and then a subtly changed environment, than would be expected by chance (Figure 1B,C). This suggests that granule cells that participate in encoding one experience are actively suppressed during the next. By contrast, there was neither a significant overlap nor suppression of granule cell activity during repeated exposures to the same environment, even when salient events such as an electric foot shock were associated with that environment (Figure 1A). This suggests that linking salient events to an environment is insufficient to drive the activation of separate populations of cells within the dentate gyrus. Importantly, the labeled cells in the dentate gyrus were not detected in the locations expected for adult-born cells, which normally reside within the inner third of the granule cell layer, suggesting that the cells activated were primarily mature granule cells. In contrast to the dentate gyrus, the CA1 subregion activated overlapping cell populations unless the mouse was placed in entirely different environments.

Overall, this study adds several important findings to our understanding of how experiences are encoded in the dentate gyrus. It shows that changes in the spatial context of an experience, but not the passage of time in the same environment nor the addition of associated fearful events, are sufficient to induce the recruitment of distinct populations of granule cells. It demonstrates that the dentate gyrus uses distinct cell populations to encode different experiences to a much greater extent than CA1, and indicates that these populations likely include large numbers of mature granule cells. Together, these results support the idea that the dentate gyrus can readily distinguish even similar experiences by coding them in separate neural populations, whereas CA1 uses largely the same cell populations and possibly alternative mechanisms to differentiate between experiences.

The results also generate new questions about how the dentate gyrus participates in memory representation. First, the conditions under which different experiences are accompanied by activation of similar populations of granule cells, as has been seen in *in vivo* electrophysiological experiments, are still largely unknown. Notably, the work of Deng et al. does not include a condition in which they were able to re-elicit activation of highly overlapping populations of granule cells. It is possible that the dentate gyrus encodes even a repetition of an event as distinct because it is, after all, a different episode. If confirmed, this would suggest a key role for the dentate gyrus in episodic memory for one-time experiences.

Moreover, the role of neurogenesis in the formation of distinct populations remains unclear. The observation that disambiguation is accomplished primarily by mature granule cells challenges the hypothesis that adult-born cells retire from the active population upon maturation (Aimone et al., 2010; Alme et al., 2010). The role of immature granule cells might contrast with that of mature cells; immature cells show heightened excitability relative to mature cells during a brief time window, meaning that multiple exposures to the same or similar environments could activate the same population of immature cells if the exposures occur within this period. While the present results leave these and many other questions to be resolved, the findings of Deng et al. suggest a new mechanism through which distinct activation patterns in the dentate gyrus might act as a driving force for identifying what is new, while CA1 tempers the news with related knowledge about the old.
